# Medical Societies in Canada

**Published:** 1865-11

**Authors:** 


Medical Societies in Canada.—Our neighbors in Canada are active in establishing medical societies. Two have recently been formed,—the "Medico-Chirurgical Society of Montreal," and the "Quebec Medical Society." Dr. George W. Campbell is President of the former, Dr. F. A. H. Lame of the latter.—*Medical & Surgical Reporter.*

